# Thermostable and Alkalistable Xylanases Produced by the Thermophilic Bacterium *Anoxybacillus flavithermus* TWXYL3

**DOI:** 10.5402/2012/517524

**Published:** 2012-09-05

**Authors:** Joshua T. Ellis, Timothy S. Magnuson

**Affiliations:** Department of Biological Sciences, Idaho State University, P.O. Box 8007, Pocatello, ID 83209, USA

## Abstract

With the rising cost and finite supply of fossil energy, there is an increasing economic incentive for the development of clean, efficient, and renewable domestic energy. The activities of microorganisms offer the potential conversion of lignocellulosic materials into fermentable sugars, usable for downstream fermentation processes. Strain TWXYL3, a thermophilic facultative anaerobe, was discovered in the Alvord Basin hydrothermal system in Oregon, USA. Phylogenetic analysis of strain TWXYL3 showed it to be 99% similar to the 16S rRNA gene of *Anoxybacillus flavithermus* WL (FJ950739). *A. flavithermus* TWXYL3 was shown to secrete a large multisubunit thermostable xylanase complex into the growth medium. Xylanase induction was achieved by resuspending the isolate in a selective xylan-containing medium. Extracellular xylanase activity showed a temperature optimum of 65°C and retained thermostability up to 85°C. Extracellular xylanase activity showed a bimodal pH optimum, with maxima at pH 6 and pH 8. Electrophoretic analysis of the extracellular xylanase shows 5 distinct proteins with xylanase activity. Strain TWXYL3 is the first xylanolytic isolate obtained from the Alvord Basin hydrothermal system and represents a new model system for development of processes where lignocellulosics are converted to biofuel precursors.

## 1. Introduction

Xylanases are enzymes that catalyze the hydrolysis of 1,4-*β*-D xylosidic linkages in xylan, the second most abundant polysaccharide in nature after cellulose, and the most abundant hemicellulose in plant cell walls [1, 2]. There has been a resurgence in interest in microbial xylanases due to their numerous uses in industrial applications, such as biobleaching of pulp [3–5] and most notably the conversion of lignocellulosic materials into fermentable substrates for production of economical and environmentally attractive biofuels [6, 7]. The complete hydrolysis of xylan involves several main-chain cleaving enzymes: endoxylanase (endo-*β*-1,4-xylanase), *β*-xylosidase (xylan 1,4-*β*-xylosidase), and *α*-glucuronidase (*α*-glucosiduronase) and side-chain cleaving enzymes: *α*-arabinofuranosidase (*α*-L-arabinofuranosidase) and acetylxylan esterase [8, 9]. Endo *β*-1,4 xylanase is one of the most common enzymes in xylan hydrolysis. This enzyme hydrolyzes the bonds between xylose subunits in the polymer of xylan to produce oligosaccharides, which in turn can be converted to xylose by *β*-xylosidase [4, 10]. 

The microbial degradation of lignocellulose is an important process because of the reliance all earth biota have on recycling of carbon and supply of both inorganic and organic carbon forms for life. The metabolic ability to recycle this carbon can be applied to creating liquid fuels, in that the sugars produced from lignocellulolysis can be converted to fuels such as ethanol and butanol. Lignocellulolytic microorganisms are ubiquitous in nature and can be isolated from plant residues such as agricultural waste products [11], or from hot spring environments where organic carbon is available [12, 13]. Members of the bacterial genera *Anoxybacillus* and *Bacillus* have been shown to secrete a variety of lignocellulolytic enzymes such as cellulases [14–16] and xylanases [6, 15–23]. These organisms produce extracellular enzymes to depolymerize hemicellulose, lignin, and cellulose present in the biosphere for a source of carbon and energy, and their xylanases are of great interest for industrial applications [6, 21]. Xylanases can be used for converting lignocellulosic agricultural waste products, which are very abundant, into fermentable sugars to generate carbon neutral liquid fuels [24, 25], as well as decreasing the amount the biologically detrimental chlorine necessary for biobleaching processes [3–5]. 

A wide variety of lignocellulolytic enzymes are commercially available; however, these enzymes are still very costly [26], due to low expression levels and the overall cost of growing the organisms to express these enzymes, consequentially limiting the efficiency of industrial-scale saccharification processes. The conversion of lignocellulosic feedstocks has been recognized as a major bottleneck in the process of biofuel production [27], due to the recalcitrant nature of plant cell walls, enzyme efficiency, and biomass quality [28]. This drives the continued discovery of novel enzymes in order to establish a better database of enzymes and identification of more efficient enzymes [29]. Additionally, the discovery of thermostable and alkalistable enzymes is desirable in order to increase catalytic efficiency throughout industrial processes due to variable conditions and treatment temperatures [6]. This study was performed to gain a better understanding of *Anoxybacillus flavithermus* TWXYL3, which was isolated from the Alvord Basin hydrothermal system in Oregon, and its extracellular xylanase enzymes.

## 2. Materials and Methods

### 2.1. Isolation and Cultivation

*A. flavithermus *TWXYL3 was isolated on Hot Springs Medium (HSM) pH 7, containing (in mg L^−1^) oat spelts xylan (7500), H_3_BO_3_ (80.1), NaCl (341.0), NaNO_3_ (4.0), KCl (54.7), K_2_HPO_4_ (100.0), MgCl_2_·7H_2_O (3.0), Wolfe's vitamin solution (10 mL L^−1^), and 100X Micronutrient solution (10 mL L^−1^), with addition of 0.5% Yeast Extract, 100X NaHCO_3_, and 100X CaCl_2_ prior to inoculation. Xylan was pretreated with 0.1 M NaOH and heated to solubilize the material prior to addition. For solid agar plates, the medium was supplemented with 15 g L^−1^ agar. Plant material on the periphery of Tinky-Winky Pool (65°C) was suspended in 10 mL of HSM containing 7.5 g L^−1^ oat spelt xylan and incubated at 65°C for 1 week. Aliquots of enrichment culture were plated onto HSM agar with oat spelt xylan. Colonies showing distinct clearing zones were transferred to fresh agar plates three times to ensure purification of the isolate. Colonies showing clearing were transferred three times to fresh agar plates. 

### 2.2. 16S rRNA Sequence Determination and Phylogenetic Analyses

Total genomic DNA was isolated from mid-log phase cultures using the DNeasy Tissue Extraction Kit (Qiagen Inc., Valencia, CA) according to the manufacturer's instructions. PCR-mediated amplification of the 16S rRNA gene was performed using primers 8F and 1492R [30] along with Platinum Taq polymerase (Invitrogen Corp., Carlsbad, CA). Sequencing of the PCR products was performed using 8F, 338F, 338R, 907F, 907R, and 1492R [30]. All DNA sequences were determined by the Idaho State University (ISU) Molecular Research Core Facility. 

The 16S sequences obtained were aligned using sequences obtained from the nonredundant NCBI database (http://www.ncbi.nlm.nih.gov) and the CLUSTALW program found in the BioEdit program (version 7.0.5.3, Department of Microbiology, North Carolina State University, Raleigh, NC). Sequences from the full-length 16S rRNA genes from multiple closely related characterized organisms resulting from BLAST, along with multiple distantly related organisms, were BLAST-searched and used in the alignment to establish relationships among TWXYL3 and phylogenetically related isolates. Eighteen sequences were used in the alignment and employed in maximum-likelihood analysis utilizing the PAUPBOOT package (Luobin Yang, Idaho State University) with 100 bootstraps. *Escherichia coli* strain KCTC 2441 (EU014689.1) was used to root the tree.

### 2.3. Enzyme Assays

Xylanase activity was measured by using Remazol Brilliant Blue dyed xylan (RBB-xylan, Sigma Chemical Co. Ltd., St. Louis, MO), which is RBB covalently coupled to 4-O-methyl-D-glucurono-D-xylan. RBB dyed xylan is a soluble chromogenic substrate that measures the breakdown of the xylan polymer by means of dye release from the substrate [31]. The substrate was dissolved in optimal reaction buffer to 10 mg/mL in 50 mM MES pH 6 for strain TWYL3 and reacted with enzyme at optimal catalytic temperature for 1 hour. The reaction was terminated with 3 volumes of 95% ethanol. After standing for approximately 5 minutes at room temperature, the precipitated substrate was removed by centrifugation at 2500 g for 5 minutes and absorbance was measured at 595 nm [17] using a Shimadzu UV-2401 PC spectrophotometer (Shimadzu Corp., Columbia, MD). One unit is defined as the amount of enzyme that catalyses the release of 1 absorbance unit of free RBB-xylan produced/minute/mg protein. These standard conditions were used for all studies. Xylanase from *Trichoderma viride* was utilized throughout as the positive control. Buffer was used in place of enzyme to serve as the negative control for all enzyme experiments.

### 2.4. Protein Determination

Protein concentration was determined using the bicinchoninic acid-copper reduction method as described by Smith et al. [22]. Briefly, 1 mL of working reagent (bicinchoninic acid/copper sulfate mixed at a 50 : 1 ratio) was added to 100 *μ*L of sample, and the absorbance was measured at 562 nm after incubating at 37°C for 30 minutes. Different concentrations of bovine serum albumin (BSA) were used as a protein standard: 0, 0.4, 0.8, 1.2, 1.6, and 2.0 mg/mL. One mL of reagent was added to 100 *μ*L BSA standardand incubated at 37°C for 30 minutes, and the absorbance was measured at 562 nm to establish a standard curve. 

### 2.5. Optimization of Xylanase Production

Xylanase production was established by growing isolates in HSM with 1% oat spelt xylan. Xylan was pretreated with 100 mM NaOH and heated to solubilize the substrate. The pH of this solution was neutralized prior to use. Cultures were grown at optimal temperature, 65°C for strain TWXYL3, and samples were assayed for xylanase activity every 24 hours to determine the optimal time for xylanase production. Contents were centrifuged at 7,500 × g for 30 min at 4°C, and the clear cell-free supernatant was filtered through a 0.22 *μ*m filter, concentrated 100-fold using a 10,000 molecular weight cut off (MWCO) membrane by means of a Millipore Amicon pressure cell (Millipore Corporation., Billerica, MA), and used as the enzyme source [6]. Enzyme preparations were assayed under standard conditions.

### 2.6. Temperature Optima and Thermostability

To determine the optimum temperature of reaction, crude enzyme preparations were assayed under standard conditions; however, the incubation temperature for the assay was varied as follows: 25°C, 37°C, 55°C, 65°C, 75°C, and 80°C. The influence of temperature on the catalytic activity of crude xylanases was determined by incubating enzyme preparations at temperatures ranging from 65°C to 95°C for times and ranging from 0 to 105 minutes without the presence of substrate. Enzyme preparations were then assayed under standard conditions. 

### 2.7. pH Optima

The effect of pH on the catalytic activity of crude xylanases was determined by assaying enzyme preparations at varying pH values ranging from 3 to 9 and assayed under standard conditions. 50 mM citric acid buffer was used for pH 3–5, 50 mM Na-acetate was used for pH 5–5.5, 50 mM MES was used for pH 5.5–6.5, 50 mM sodium phosphate was used for pH 6.5–7.5, and 50 mM Tris-HCl was used for pH 7.5–9. Crude preparations were adjusted to the appropriate pH by means of buffer exchange. Additionally, the pH of the buffers were adjusted under the standard catalytic temperature to ensure accurate pH values throughout. Enzyme preparations were then assayed under standard conditions.

### 2.8. Xylanase Purification

Crude preparations were initially concentrated 100-fold using a Millipore Amicon pressure cell (Millipore Corp., Billerica, MA) with a 10,000 MWCO membrane. Partial purification of xylanase enzymes from strain TWXYL3 was achieved by use of a HiPrep 26/60 Sephacryl S-200 high-resolution size exclusion column (Amersham Biosciences Corp., Piscataway, NJ). The column was equilibrated with 50 mM sodium phosphate buffer, pH 7.2, containing 150 mM NaCl. Active xylanase fractions were then loaded onto a HiPrep desalting column (Amersham Biosciences Corp., Piscataway, NJ) equilibrated with 50 mM Tris-HCl, pH 8.45. This preparation was then loaded onto a HiPrep 16/10 Q FF ion exchange column (Amersham Biosciences Corp., Piscataway, NJ) equilibrated with 50 mM Tris-HCl, pH 8.45. Bound proteins were eluted with a linear gradient of 0.0–1.0 M NaCl in 50 mM Tris-HCl, pH 8.45. The xylanase activity eluted at 0.2 M NaCl.

### 2.9. Electrophoresis and Zymography

A 5.0% Tricine nondenaturing (ND) polyacrylamide gel electrophoresis (PAGE) system was utilized in order to obtain enzyme profiles for extracellular fractions in their native, multimeric state [32]. Approximately 50 *μ*g of protein was loaded and electrophoresed at 30 mA/gel constant current at 4°C. Gels were stained for xylanase activity (described below) and/or stained overnight in Coomassie Brilliant Blue (CBB) and destained in deionized water. Gel images were then captured and digitized using VersaDoc 3000 imaging system (Bio-Rad Laboratories, Inc., Hercules, CA). A 7.5% Tricine sodium dodecyl sulfate (SDS) PAGE system was utilized in order to obtain enzyme profiles in their monomeric state [32]. Approximately 50 *μ*g of protein was denatured, loaded, and electrophoresed at 30 mA/gel constant current. Gels were dyed overnight in CBB and destained in deionized water. Gel images were then captured and digitized as described above.

Zymogram analysis was performed after SDS-PAGE or ND-PAGE as described by Matsui et al. [34] with some minor modifications. Briefly, samples were treated as described above and loaded onto a 7.5% Tricine SDS-PAGE or 5.0% Tricine ND-PAGE [32] containing 0.15% xylan. ND-PAGE zymograms were run in a cold room set at 4°C. After electrophoresis, the proteins were renatured (if applicable) in 100 mM sodium phosphate buffer (pH 6.8) containing 2% Triton X-100 for 30 minutes with mild shaking. Gels were then incubated in 100 mM sodium phosphate (pH 6.8) buffer for 1 hour at optimal enzyme temperature with gentle shaking. Gels were stained using 0.1% Congo red for 10 minutes, destaining with 1% NaCl until zones of clearing became present, and fixed with 5% acetic acid. Zones of clearing correspond to enzyme activities on xylan. The gel images were then captured and digitized as described above. 

## 3. Results 

### 3.1. Isolation and Phylogenetic Characterization of Strain TWXYL3

**Figure 1:**
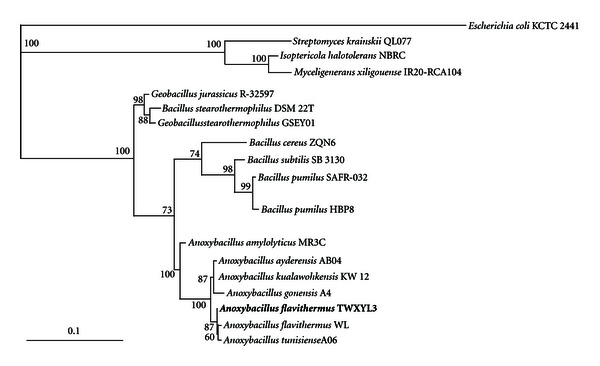
Phylogenetic tree illustrating position of strain TWXYL3, based on maximum likelihood analysis using PAUPBOOT package in EGG software with 100 bootstraps. Scale bar corresponds to 0.1 substitutions per nucleotide position.

Strain TWXYL3 was obtained from submerged plant material on the periphery of a stagnant 55°C pool in the Mickey Hot Springs area of the Alvord Basin. Isolation was achieved by enrichment on xylan-containing synthetic hot spring medium, followed by successive streak isolation to obtain a pure culture. TWXYL3 was judged pure by 16S rRNA gene sequence analysis, and consistent colony and cellular morphology. Analyses of pure culture isolate DNA did not show multiple 16S rRNA gene sequences, indicating that the culture was pure. The partial 16S rRNA sequence was obtained with a length of 1,382 bps. Analysis of the 16S rRNA gene showed that strain TWXYL3 had high similarity to the genus *Anoxybacillus*. Phylogenetic analysis of 16S rRNA from strain TWXYL3, along with additional similar and dissimilar characterized strains, was utilized to construct a phylogenetic tree (Figure 1).

### 3.2. Optimization of Xylanase Production

**Figure 2:**
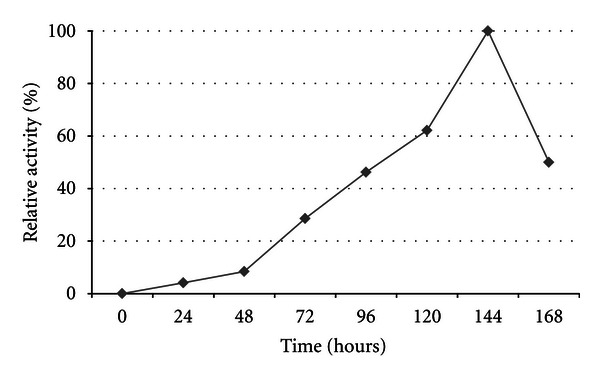
Relative total xylanase activity from strain TWXYL3 grown in HSM with 1.0% oat spelt xylan. The specific activity of the xylanase enzymes from the crude supernatant was measured at 0.08 U/mg protein at day 6 (144 hours). The values are means of three replicates at each temperature.

Cultures were grown with 1% oat spelt xylan, and the optimal incubation time was determined for xylanase production. Spectrophotometric assays show the optimal enzyme temperature and incubation time for strain TWXYL3 to be 65°C for approximately 6 days (Figure 2). The specific activity of the xylanase enzymes from the crude supernatant was measured at 0.08 U/mg protein.

### 3.3. Characterization and Partial Purification of Extracellular Xylanases

**Figure 3:**
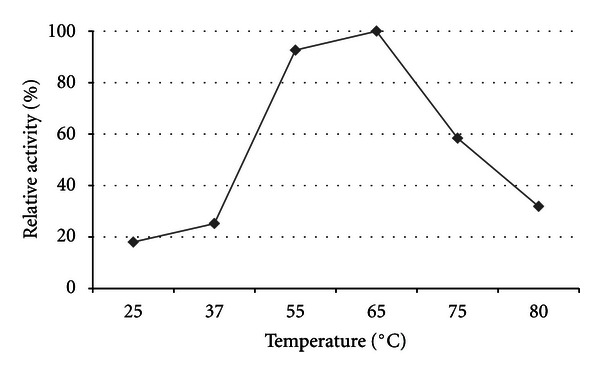
Optimal temperature of xylanases from strain TWXYL3. The values are means of three replicates at each temperature.

**Figure 4:**
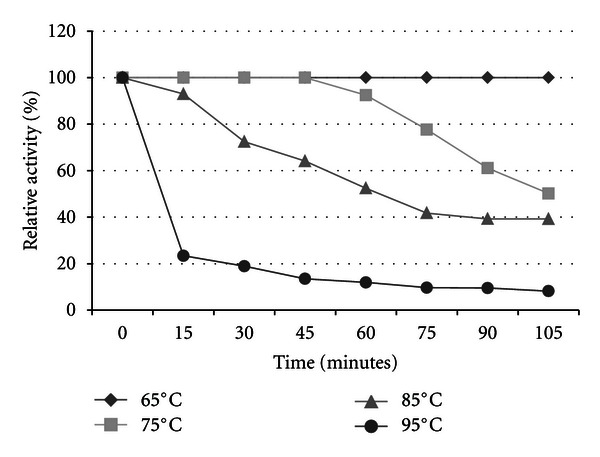
Thermostability of crude xylanase activities from strain TWXYL3. Activity was measured using standard conditions after time intervals indicated. The values are means of three replicates at each time point.

**Figure 5:**
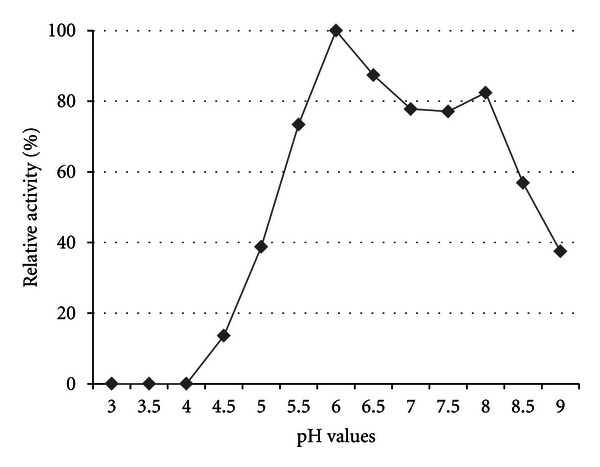
Effect of pH on crude xylanase activity from strain TWXYL3. Values are the mean of three replicates at each pH. Assay was conducted using standard conditions; however, the pH was varied as indicated in the figure.

The xylanase activities produced by strain TWXYL3 were highest between 55°C and 65°C, with optimal activity at 65°C (Figure 3). Although the stability of these enzymes were significantly reduced at 85°C, showing 52% activity after 60 minutes and 39% activity after 105 minutes, they retained 92% activity at 75°C after 60 minutes (Figure 4). Xylanase enzymes were optimal at pH 6; however, they retained 38% of their activity at pH 9 for one hour (Figure 5). The specific activity of the xylanase enzymes from the crude supernatant was measured at 0.08 U/mg protein and 2.2 U/mg protein after partial purification.

### 3.4. Electrophoretic and Zymographic Analysis

**Figure 6:**
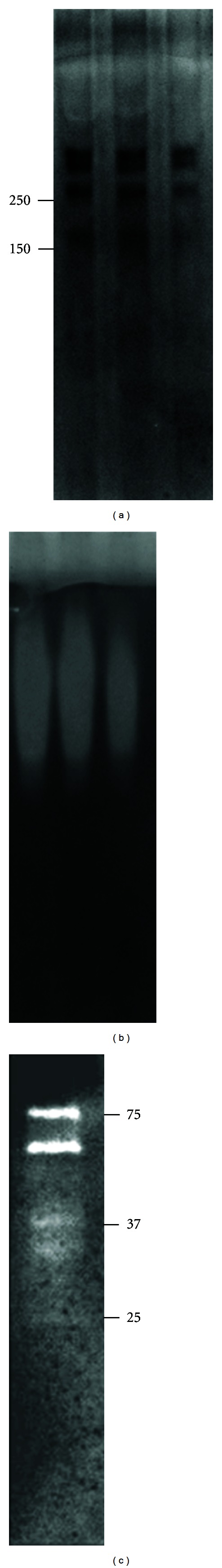
5.0% Tricine ND-PAGE and ND-zymogram analysis containing 0.15% xylan, using crude xylanase preparations from strain TWXYL3, depicting the presence of high-molecular-weight proteins and high-molecular-weight active xylanases. Molecular mass markers are Precision Plus Protein Standards (Bio-Rad). (a) ND-PAGE analysis of crude enzyme preparations (50, 25, and 10 *μ*g loading), stained with CBB. (b) Zymogram analysis of high-molecular-weight crude xylanase enzymes (50, 25, and 10 *μ*g loading). Xylanase activity is indicated by zones of clearing. (c) A 7.5% Tricine SDS-PAGE zymogram containing 0.15% oat spelt xylan as substrate, using crude xylanase preparation (50 *μ*g) from strain TWXYL3. Xylanase activity is indicated by zones of clearing.

ND-PAGE and zymography assays from crude extracellular preparations indicate the presence of a large-molecular-weight active xylanase complex equal to or greater than 250 kDa. Despite performing the experiment at 4°C, activity was present even during electrophoresis, as evidenced by smeared clearing zones or tracks (Figure 6). Additionally, SDS-PAGE zymography assays indicated multiple xylanase activity bands ranging from 25 to 75 kDa, with activity bands present at 25 kDa, 32 kDa, 37 kDa, 60 kDa, and 75 kDa, also produced from strain TWXYL3 (Figure 6).

## 4. Discussion

TWXYL3 is a facultative anaerobic thermophile that secretes moderately alkalistable and thermostable xylanase enzymes into the growth medium. Phylogenetic analysis supports the relationship of strain TWXYL3 to other characterized strains of *A*. *flavithermus*.  Table 1 shows general characteristics of *A. flavithermus *TWXYL3 as well as other similar characterized strains. Our isolate was able to grow on a wide variety of carbohydrates, including dairy waste as the sole carbon source. This ability to degrade an abundance of lignocellulosic materials would be advantageous for industrial-scale saccharification processes.

Only one other known strain of *A. flavithermus *has shown xylanolytic capabilities [15], and among the genus *Anoxybacillus*, only the species of *Anoxybacillus flavithermus* has shown capable of xylan and cellulose-degrading capabilities [27]. Xylanase enzymes described here have been shown to be thermostable as well as moderately alkalistable. The thermostability of these enzymes was similar to published results of *A. flavithermus *BC by Kambourova et al. [15], in that these xylanases were highly stable at temperatures around 70°C. An earlier study of *A. flavithermus *BC by the same group provided similar results on the alkalistability of *A. flavithermus *xylanase enzymes [35]. 

Partial purification of xylanase enzymes indicates that there was measurable and increased xylanase activity throughout purification of these enzymes. This indicates that these enzymes could be purified and employed in industrial processes. Specific activity of xylanase enzymes from strain TWXYL3 throughout enzyme purification was measured at 0.08 U/mg protein in the crude supernatant and 2.2 U/mg protein after concentration, size exclusion, and anion exchange chromatograph was employed.

These data illustrate that *A. flavithermus *TWXYL3 shows interesting xylanolytic capabilities in that it secretes a large pentameric enzyme complex into the growth media, which has never been described by this species. This complex, or xylanosome, is composed of at least 5 active protein subunits ranging from 25 kDa to 75 kDa, suggesting a pentameric protein complex approximately 250 kDa in size (Figure 6). This data is unique from the xylanase activity described for Strain BC [15], in that they illustrated only two active xylanase enzymes at 92 kDa and 80 kDa. The presence of a large-molecular-weight active complex from strain TWXYL3 is further supported by the elution profile from the S-200 high-resolution size exclusion column (data not shown). The active fractions are eluted in the area of a 200 kDa or larger protein when compared to standards of greater than 200 kDa. Thermophiles typically produce family 10 (large molecular mass) and family 11 (small molecular mass) xylanases [36], and the profile of xylanase proteins detected in TWXYL3 is consistent with this pattern. 

The ability of *A. flavithermus *TWXYL3 to produce a large xylanolytic complex may serve to provide higher adaptability to degrade an assortment of complex polysaccharides or perhaps to fully degrade xylan into its monosaccharide components. Thermostable xylanases from microorganisms provide great benefit in that they can retain their activities at high temperatures, while preventing potential contamination due to these high temperatures [37]. Additionally, thermostable enzymes simplify cooling problems from pretreated biomass [37, 38]. Xylanases described here would be ideal for certain industrial applications [2, 27], particularly biobleaching of pulp [6, 18] and saccharification processes [6] due to their adaptive capacity to withstand hostile conditions. This microorganism and its extracellular xylanases show potential in large scale-up reactors, for the conversion of agricultural waste products rich in lignocellulose, which are cheap and abundant, for use in downstream fermentation processes into high-value biofuels.

## Figures and Tables

**Table 1:** General characteristics of *A.* *flavithermus* TWXYL3, *A.* *flavithermus* DSM 2641, *Anoxybacillus* *ayderensis* ABO4, and *Anoxybacillus* *kestanbolensis* K4 [33].

	TWXYL3	DSM 2641	ABO4	K4
Morphology	Bacilli	Bacilli	Bacilli	Bacilli
Gram reaction	Gram positive	Gram positive	Gram positive	Gram positive
Optimal growth temperature (°C)	65	60–65	50–55	50–55
Temperature growth range (°C)	35–75	30–72	30–70	40–70
Optimal growth pH	7.0	7.0	7.5–8.5	7.5–8.5
pH growth range	6.0–10.0	5.5–9.0	6.0–11.0	6.0–10.5
Salt tolerance	3% NaCl	2.5%	2.5%	4.0%
Aerotolerance	Facultative	Facultative	Facultative	Facultative
Motility	+	+	+	+
Spore formation	+	+	+	+
Biofilm formation	+	ND	ND	ND
Hydrolysis of				
birchwood xylan	+	ND	ND	ND
beechwood xylan	+	ND	ND	ND
oat Spelt xylan	+	ND	ND	ND
dairy waste	+	ND	ND	ND
Growth on		ND		
cellulose	+	ND	ND	ND
glucose	+	ND	+	+
L-arabinose	+	+	+	+
D-fructose	+	ND	+	+
D-xylose	+	−	+	+
D-galactose	+	ND		
D-mannose	+	+	+	+
D-Lactose	+	−	−	−
starch	+	ND	+	+
sucrose	+	ND	+	+
